# Cerebral vasospasm following arteriovenous malformation rupture: a population-based longitudinal study

**DOI:** 10.1007/s00701-026-06816-4

**Published:** 2026-03-18

**Authors:** Isaac B. Thorman, Eris Spirollari, Tamer Mubarak, Eman Elbayoumi, Aryan Malhotra, Ariel Sacknovitz, Ankita Jain, Michael C. Schubert, Uchenna Okafo, Gurmeen Kaur, Andrew Bauerschmidt, Jon Rosenberg, Stephan A. Mayer, Chirag D. Gandhi, Fawaz Al-Mufti

**Affiliations:** 1 School of Medicine, New York Medical College, 40 Sunshine Cottage Rd, Valhalla, NY USA; 2 Department of Otolaryngology – Head and Neck Surgery, Johns Hopkins University, Baltimore, MD USA; 3 Department of Neurosurgery, Westchester Medical Center, 100 Woods Road, Valhalla, NY 10595 USA; 4 Dubai Academic Health Corporation, Dubai, UAE; 5 Department of Neurobiology, Center of Neurotrauma, Multi-Omics & Biomarkers (CNMB), Neuroscience Institute, Morehouse School of Medicine, Atlanta, GA USA; 6 Department of Physical Medicine and Rehabilitation, Johns Hopkins University, Baltimore, MD USA; 7 Department of Neurology, Westchester Medical Center, Valhalla, NY USA

**Keywords:** Intracranial Arteriovenous Malformations, Vasospasm, Intracranial, Intracranial Hemorrhages, Critical Care

## Abstract

**Background:**

Vasospasm is a devastating sequelae of ruptured arteriovenous malformations (AVMs) in adults. Comorbidities and presenting factors have been suggested as risks, but only in cross-sectional studies. The objective of this study was to characterize risk factors associated with vasospasm and mortality in ruptured AVM.

**Methods:**

Adult patients from the TriNetX Research Network were included, based on the ICD-10 codes, over a period of 20 years. Cox proportional hazard models assessed the hazards of vasospasm (I67.84) and mortality separately, adjusting for age, sex, comorbidities, substance use history, presenting factors (e.g., hypernatremia, hypokalemia), criteria of the National Inpatient Sample-Subarachnoid Hemorrhage Severity Score, and location of hemorrhage. Outcomes were assessed in the first 30 days following rupture.

**Results:**

Among 10,375 patients with ruptured AVMs, 523 (5.3%) died and 297 (3.0%) experienced vasospasm in the first 30 days. After matching for age and sex, vasospasm was associated with increased mortality at three months (11.1% vs. 4.8%, *p* = 0.003), six months (12.6% vs. 5.1%, *p* = 0.001), and one year (13.5% vs. 6.9%, *p* = 0.005). Female sex was protective against vasospasm within 30 days (HR = 0.714, *p* = 0.007) while the greatest risk factors present on admission included subarachnoid hemorrhage (6.086, *p* < 0.001), hydrocephalus (3.783, *p* < 0.001), and leukocytosis (2.0677, *p* < 0.001). The greatest risk factors for 30-day mortality were coma (HR = 3.700, *p* < 0.001), hydrocephalus (2.698, *p* < 0.001), and chronic kidney disease (1.596, *p* = 0.003).

**Conclusions:**

In this retrospective cohort study of 10,375 adult patients with ruptured AVMs, vasospasm occurred in approximately 3%. Risk factors for vasospasm included subarachnoid hemorrhage, male sex, hydrocephalus, and leukocytosis. The presence of vasospasm was associated with a more than twofold increase in mortality at both 30 days and one year.

## Introduction

Cerebral arteriovenous malformations (AVMs) are congenital vascular anomalies with a high risk of rupture, resulting in high rates of morbidity and mortality [5]. Hemorrhagic events often result in secondary consequences such as hydrocephalus, elevated intracranial pressure, and cerebral vasospasm. Vasospasm is a leading cause of cerebral ischemia; its sequelae are well-studied and documented throughout various cerebrovascular disease states [5, 7, 13, 21]. However, the incidence and risk factors of vasospasm after AVM rupture are still poorly documented [11].

There are conflicting estimates of vasospasm occurrence, ranging from 1.1% to 17%, depending on study methodology and diagnostic criteria [21]. A study using the National Inpatient Sample (2015–2019 data) showed that at least 13% of patients develop vasospasm following AVM rupture. In contrast, single-institution studies report lower prevalences of vasospasm, including as a review of 84 hemorrhagic events in 73 AVM patients in which only one case (1.1%) of mild angiographic vasospasm was documented [14].

Identification and mitigation of risk factors for vasospasm after AVM rupture may help avoid the sequelae of vasospasm. Multiple previous studies have linked the risk of vasospasm after intracranial hemorrhage rupture to female sex, younger age, intraventricular hemorrhage, smoking, chronic hypertension, hyponatremia, leukocytosis, and acute hypotension [1, 7, 10, 36]. Notably, vasospasm has been linked to a higher risk of delayed cerebral ischemia, which lowers the chance of routine discharge and dramatically increases in-hospital mortality [10]. However, the majority of published evidence regarding vasospasm in intracranial hemorrhages has been determined in aneurysmal or traumatic subarachnoid hemorrhage. As a result, management of patients with ruptured AVM has been extrapolated from these other disease states.

The body of literature on vasospasm following AVM rupture is still mostly limited to case reports, small institutional series, and cross-sectional analyses. To close this gap, we are aiming to establish a comprehensive, population-based evaluation of vasospasm in this setting to better understand its incidence, risk factors, and clinical impact. In our study, we leveraged a large-scale, longitudinal dataset from the TriNetX Research Network to characterize the risk factors for vasospasm in ruptured AVMs. Additionally, we sought to re-assess risk factors for vasospasm in patients with ruptured AVMs using a longitudinal lens.

## Methods

This retrospective, longitudinal cohort study sourced patient data from the TriNetX Research Network, a federated dataset which provided access to the deidentified, longitudinal, electronic medical records (diagnoses, procedures, medications, laboratory values) of approximately 151 million patients from 107 healthcare organizations [26]. Of these healthcare organizations, 55 are academic, 44 are non-academic, and eight are an unknown type. Electronic medical record data are collected as part of routine clinical care in both inpatient and outpatient settings, and data are retrieved in a structured manner for demographics, diagnoses, procedures, medications, laboratory tests, and vital signs using accepted coding methodology. Elements of narrative text from clinical documents were extracted by TriNetX using a proprietary Natural Language Processing program [34]. As data were collected for clinical purposes, “missing data” is interpreted as suggesting that a patient did not have the event of interest [34, 35]. This is a secondary analysis of existing data which does not involve intervention or interaction with human subjects, and is de-identified per the standard defined in Section §164.514(a) of the HIPAA Privacy Rule. Therefore, this study is exempt from Institutional Review Board/ethics committee approval and individual patient consent. Updates and new patients are added daily, so the number of patients present at the moment of each individual analysis may fluctuate.

Within the Network, adult patients (≥ 18 years) were included if they had a diagnosis of an AVM (Q28.2 or Q28.3) on the day of an intracranial hemorrhage (I60-I62). The diagnostic codes for AVM have been previously published in database-type studies [2, 27, 28]. Patients were indexed on the day of their hemorrhage. Patients were followed for up to one year, the first instance of vasospasm (I67.84), the end of the medical record, or mortality. TriNetX acquires mortality data from healthcare organization death records, billing codes, the Social Security Administration Master Death File, private obituaries, and private claims [34].

Statistical analyses were performed using TriNetX’s Advanced Analytics Platform, using R version 3.4.4 (R Foundation for Statistical Computing, Vienna, Austria), and Python version 3.6.5 (Python Software Foundation, Centrum voor Wiskunde en Informatica, Amsterdam, The Netherlands) [17]. Statistical significance was established at α < 0.01. We used 1:1 propensity score matching based on age and sex to assess the risk of mortality associated with vasospasm. A nearest-neighbor method was utilized with a caliper of 0.1 pooled standard deviations and without replacement [34]. We assessed the hazards of vasospasm and mortality using separate multivariate Cox proportional hazard models. In line with prior literature [10, 38], we controlled for sex, age, comorbidities (e.g., diabetes, chronic hypertension, obesity, heart failure), substance use disorders (nicotine, alcohol, cocaine), hospitalization events (e.g., leukocytosis, hyper/hyponatremia, hyper/hypokalemia), physical exam findings (e.g., paralytic strabismus, stupor), interventions (e.g., ventriculostomy, intubation), and type of hemorrhage (e.g., subarachnoid, subdural, extradural).

## Results

We included 10,375 adult patients with a ruptured brain AVM. The mean age at rupture was 51.9 years (SD = 17.5 years), 48% identified as female, 70% identified as non-Hispanic or Latino ethnicity, 61% identified as White race, 12% identified as Black race, and 8% identified as Asian race. Prior to the day of rupture, prevalent cardiovascular comorbidities included chronic hypertension (29%), hyperlipidemia (23%), chronic ischemic heart disease (9%), atrial fibrillation and flutter (5%), heart failure (6%), and atherosclerosis (4%). Likewise, neurological comorbidities included sleep disorders (13%), pain (12%), and epilepsy (11%). The prevalence of vasospasm was 3% and the prevalence of mortality was 5%. While there was no significant difference in 30-day mortality by vasospasm (6.8% vs. 4.9%, *p* = 0.144), the difference fell short of significant after 1:1 propensity score matching based on age and sex (7.8% vs. 4.2%, *p* = 0.0504). After controlling for confounding variables, vasospasm was associated with increased mortality at three months (11.1% vs. 4.8%, *p* = 0.003), six months (12.6% vs. 5.1%, *p* = 0.001), and one year (13.5% vs. 6.9%, *p* = 0.005).

**Fig. 1:**
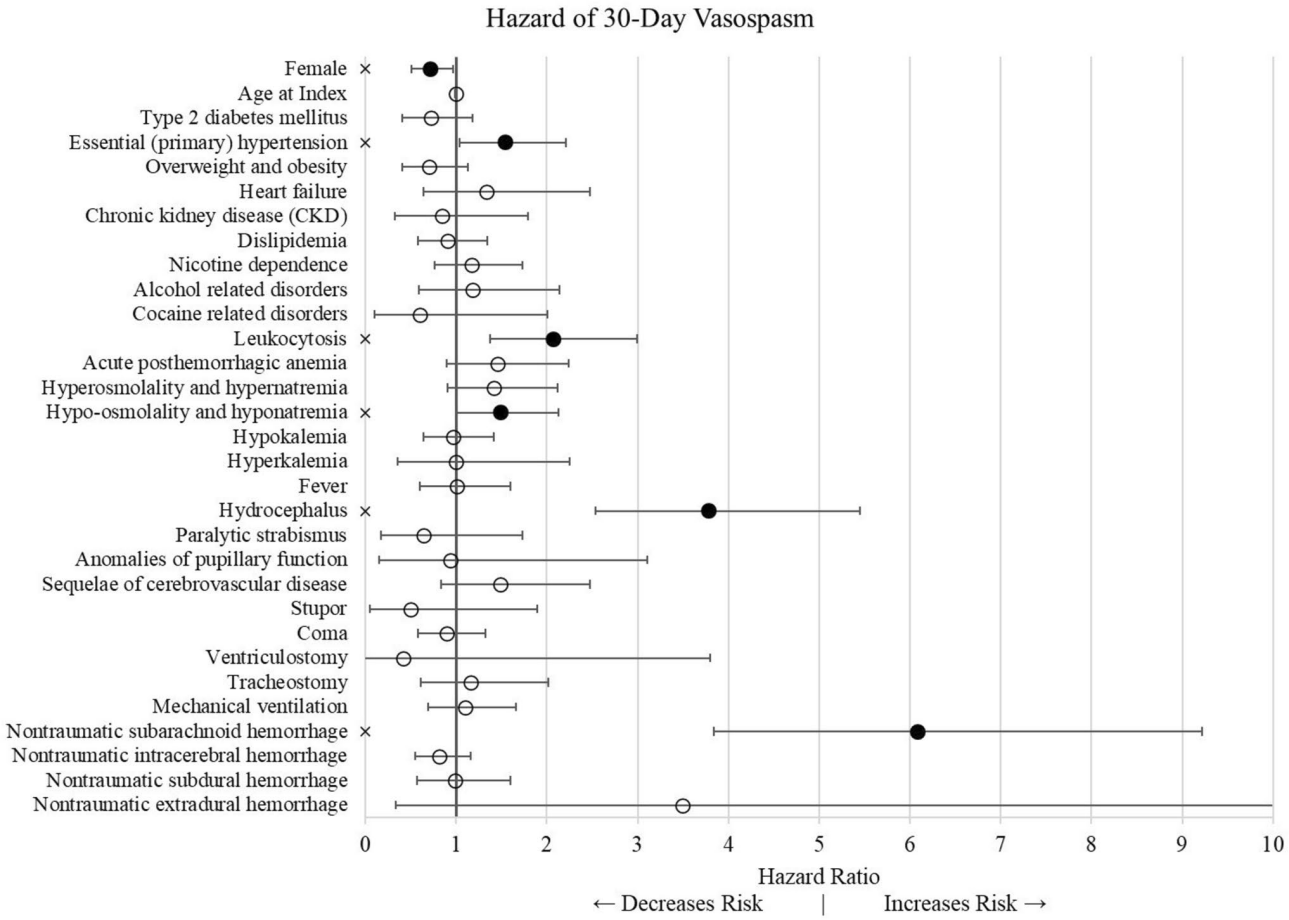
Risk factors for vasospasm in 30 days following AVM rupture. Error bars represent the 99% confidence interval. ✕ indicates significance at a 99% confidence level. The upper limit of the confidence interval for nontraumatic extradural hemorrhage is 13.587

We used a multivariate Cox proportional hazard model to assess for the risk of vasospasm within 30 days of rupture (Fig. 1). Female sex was protective against vasospasm (HR = 0.714; 95% CI: 0.559, 0.910; *p* = 0.007) while a history of hypertension was associated with an increased risk (HR = 1.544; 95% CI: 1.162, 2.051; *p* = 0.003). Hospitalization events associated with an increased risk of vasospasm included leukocytosis (HR = 2.067; 95% CI: 1.540, 2.775; *p* < 0.001), hyponatremia (HR = 1.489; 95% CI: 1.120, 1.978; *p* = 0.006), and hydrocephalus (HR = 3.783; 95% CI: 2.832, 5.054; *p* < 0.0001). Subarachnoid (HR = 6.086; 95% CI: 4.374, 8.467; *p* < 0.0001) hemorrhage type was associated with increased risks of vasospasm.

**Fig. 2:**
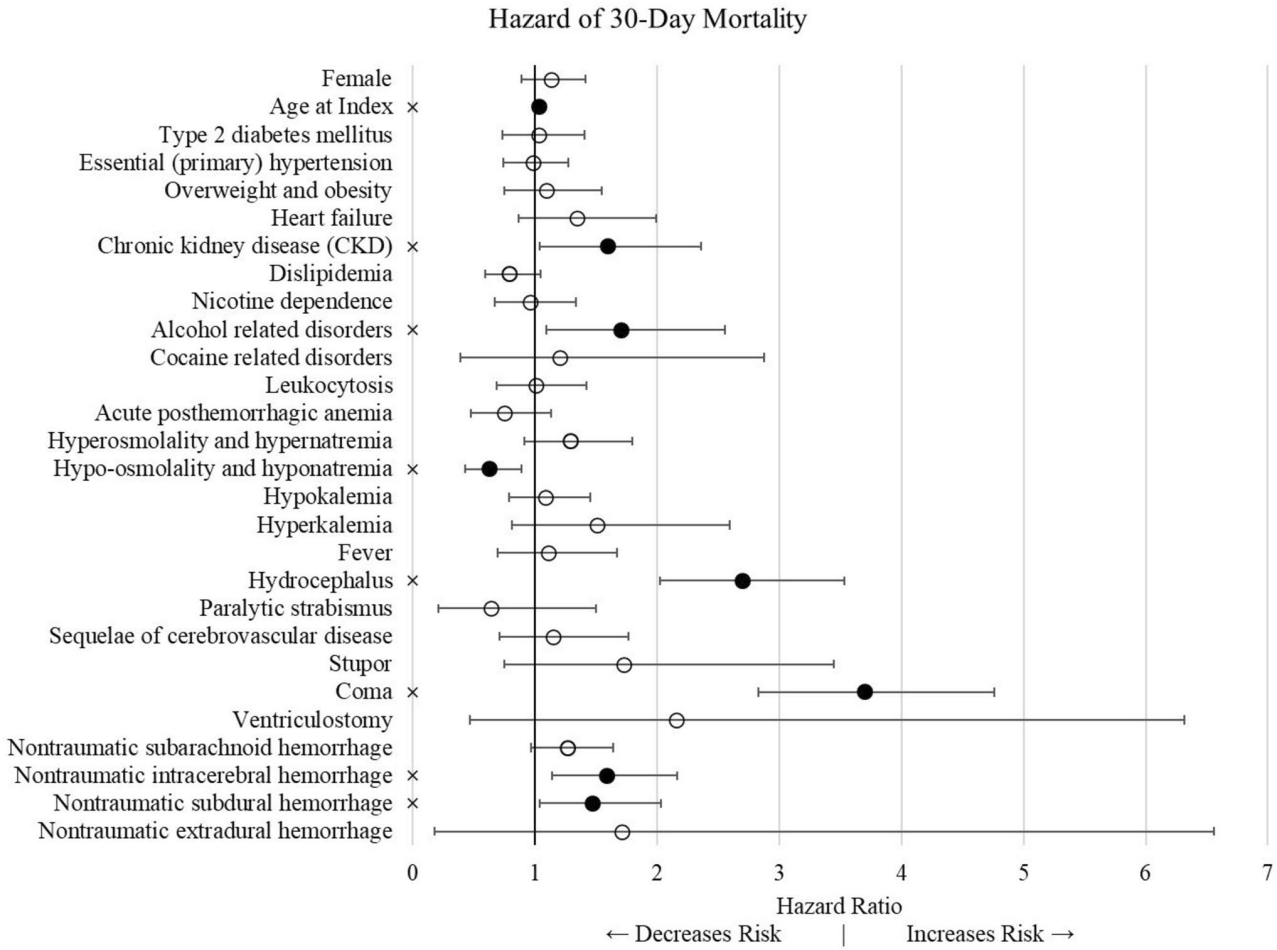
Risk factors for mortality in 30 days following AVM rupture. Error bars represent the 99% confidence interval. ✕ indicates significance at a 99% confidence level

Likewise, a multivariate Cox proportional hazard model assessed for the risk of mortality within 30 days of rupture (Fig. 2). Increased age at rupture (HR = 1.037; 95% CI: 1.030, 1.043; *p* < 0.001) and alcohol use disorder (HR = 1.707; 95% CI: 1.240, 2.350; *p* = 0.001) were associated with increased mortality. Factors present on admission such as hydrocephalus (HR = 2.698; 95% CI: 2.183, 3.334; *p* < 0.001) and coma (HR = 3.700; 95% CI: 3.037, 4.507; *p* < 0.001) were associated with increased risks of mortality, while hyponatremia (HR = 0.627; 95% CI: 0.476, 0.825; *p* = 0.001) was associated with decreased mortality. Intracerebral (HR = 1.589; 95% CI: 1.244, 2.028; *p* < 0.001) and subdural (HR = 1.471; 95% CI: 1.139, 1.899; *p* = 0.003) hemorrhage types were significant risk factors for mortality.

## Discussion

In this retrospective, longitudinal, population-based study, the risk of vasospasm in the 30 days following an AVM rupture was 3.0% while 30-day, all-cause mortality was 5.0%. After matching on age and sex, vasospasm was a significant risk factor mortality at three months, six months, and one year. Although this falls below the 13.9% prevalence of angiography-confirmed vasospasm shown by an institutional review of 36 patients with ruptured AVM [5], prior literature has reported prevalences ranging widely between 2 and 31% [10]. However, as we present an analysis of real-world data, our estimated prevalence of vasospasm is likely reflective of clinical vasospasm, as opposed to angiographic or radiographic vasospasm. Prior works have shown that only 20–30% of patients with clinical vasospasm will show clinically significant neurological deficits [12, 19, 39]. Therefore, as only 3.0% of patients demonstrated clinical signs and symptoms suggestive of vasospasm, it stands to reason that 10–15% of our cohort may have experienced angiographic vasospasm.

We found that chronic hypertension was a significant risk factor for subsequent vasospasm, but female sex appeared to be protective. In previous studies of aneurysmal subarachnoid hemorrhage, chronic hypertension was shown to increase the risk of post-hemorrhagic vasospasm [10, 16], though the mechanism underlying this remains unclear. It is speculated that underlying atherosclerotic disease may reduce the inherent ability of blood vessels to withstand hypoxia, reducing the threshold for vasospasm [20, 25]. In contrast to our results, prior studies have consistently shown that female sex is associated with increased risk of vasospasm after both aneurysmal and AVM rupture [7, 8, 10, 13, 29]. The reasons for this disagreement are unknown but may relate to the multivariate adjustment of the present analysis as opposed to the univariate adjustments previously published. There may also exist a survivorship bias, in which female patients survived the initial bleed.

Risk factors for cerebral vasospasm present on admission included leukocytosis, acute posthemorrhagic anemia, hydrocephalus and electrolyte disturbances. These findings have been demonstrated previously in the National Readmission Database, in which anemia, hypovolemia, hypotension, leukocytosis, hyponatremia, and hypokalemia were associated with increased vasospasm risk after ruptured aneurysms [31]. These factors were similarly shown to be associated with increased risk following vasoin the National Inpatient Sample [10]. Mechanistically, long-standing evidence has suggested that disruptions in effective circulating volume by posthemorrhagic anemia, hypovolemia, and hypotension may predispose patients to vasospasm [30, 37]. Prior evidence suggests that this relationship may be attributed to a dysfunctional compensatory cerebral autoregulation mechanism leading to hypoxic neuronal injury [9, 18, 24]. Though calcium has long been implicated in the pathogenesis of vasospasm, and calcium channel blockers may be used in treatment of vasospasm, it is unclear whether other electrolyte abnormalities are causatively involved in vasospasm or reflective of underlying volume status changes [3, 4, 6, 15, 22, 23, 32, 40].

While our results largely reaffirm those previously shown, the present analysis extends prior work, including a nationally-representative, cross-sectional analysis, in several respects [10]. The superior sample size and diversity, inclusive of both inpatient and outpatient encounters as well as data from multiple countries, increases the external validity. Longitudinal data showed that patients with vasospasm experienced twice the risk of mortality through the first year after AVM rupture compared to patients who did not have vasospasm. Out-of-hospital mortality was captured in addition to in-patient mortality, which has been an outcome of interest previously. Further, longitudinal data enables us to assess the temporal sequence of events. We now present more granular medical record data regarding patient management which were previously unavailable, such as ventriculostomy and mechanical ventilation.

Our study is limited by its retrospective design and reliance on administrative ICD-10 code, which may misclassify or bias diagnoses. Under-reporting of specific conditions and interventions is a known hazard when using administrative databases. Thus it is possible that the 3.0% frequency of vasospasm that we found is lower than might have been found had systematic angiography been performed. Although diagnostic cerebral angiograms were not captured, it remains the gold standard for detecting vasospasm and associated mortality risk in ruptured AVMs [41]. Diagnostic codes for AVM (Q28.2 and Q28.3) have been previously published in studies using similar methodology, but we could not eliminate the possibility that other types of cerebral vascular malformations were included in our study population. Our findings may also have been swayed by aneurysms associated with AVMs as we were not able to exclude these using ICD-10 codes. Diagnoses, procedures, and laboratory tests were ascertained through the medical record, as opposed to patient- or physician-reported data, although self-report measures come with their own biases. Updates and new patients are added constantly to the TriNetX Advanced Analytics Platform, and servers are periodically unreachable, so the number of patients present at the moment of each individual analysis may fluctuate. As has been previously published [17, 33, 34], we were unable to access granular information regarding the specialty of the consulting physician, which guidelines were followed, or what the severity of the condition was at the time of diagnosis. Given the size of the network (approximately 151 million patients across 107 healthcare organizations in multiple countries), heterogeneity of clinical practice was inevitable between and within healthcare organizations, including missing data, differences in diagnostic criteria, insurance coverage, and inpatient vs. outpatient differences in coding and recording.

## Conclusion

Vasospasm is a well-known complication of ruptured AVM. In this retrospective, longitudinal cohort study, significant factors associated with vasospasm included male sex, hypertension, hydrocephalus, subarachnoid hemorrhage, leukocytosis, anemia, hypernatremia, and hyponatremia. There has only been one prior cohort study of vasospasm after ruptured AVM, and this used a similar database methodology. Prospective studies with analysis of radiographic images are critically needed in identifying and managing patients at risk of post-rupture vasospasm.

## Data Availability

Data not available due to commercial restrictions. The data that support the findings of this study are available from TriNetX, LLC but third-party restrictions apply to the availability of these data. The data were used under license for this study with restrictions that do not allow for the data to be redistributed or made publicly available. However, for accredited researchers, the TriNetX data is available for licensing at TriNetX, LLC. Data access may require a data sharing agreement and may incur data access fees.

## References

[1] Al-Mufti F, Amuluru K, Lander M, Mathew M, El-Ghanem M, Nuoman R, Park S, Patel V, Singh IP, Gupta G, Gandhi CD (2018) Low Glasgow Coma Score in traumatic intracranial hemorrhage predicts development of cerebral vasospasm. World Neurosurg 120:e68–e71. 10.1016/j.wneu.2018.07.143 30055364 10.1016/j.wneu.2018.07.143

[2] Al-Shahi R, Bhattacharya JJ, Currie DG, Papanastassiou V, Ritchie V, Roberts RC, Sellar RJ, Warlow CP (2003) Scottish Intracranial Vascular Malformation Study (SIVMS). Stroke 34:1156–1162. 10.1161/01.STR.0000069012.23858.69 12702840 10.1161/01.STR.0000069012.23858.69

[3] Allen GS (1985) Role of calcium antagonists in cerebral arterial spasm. Am J Cardiol 55:149b–153b. 10.1016/0002-9149(85)90624-1 3881907 10.1016/0002-9149(85)90624-1

[4] Allen GS, Ahn HS, Preziosi TJ, Battye R, Boone SC, Boone SC, Chou SN, Kelly DL, Weir BK, Crabbe RA, Lavik PJ, Rosenbloom SB, Dorsey FC, Ingram CR, Mellits DE, Bertsch LA, Boisvert DP, Hundley MB, Johnson RK, Strom JA, Transou CR (1983) Cerebral arterial spasm–a controlled trial of nimodipine in patients with subarachnoid hemorrhage. N Engl J Med 308:619–624. 10.1056/nejm198303173081103 6338383 10.1056/NEJM198303173081103

[5] Amuluru K, Al-Mufti F, Romero CE, Gandhi CD (2018) Isolated intraventricular hemorrhage associated with cerebral vasospasm and delayed cerebral ischemia following arteriovenous malformation rupture. Interv Neurol 7:479–489. 10.1159/000490583 30410528 10.1159/000490583 PMC6216786

[6] Barth M, Capelle HH, Weidauer S, Weiss C, Münch E, Thomé C, Luecke T, Schmiedek P, Kasuya H, Vajkoczy P (2007) Effect of nicardipine prolonged-release implants on cerebral vasospasm and clinical outcome after severe aneurysmal subarachnoid hemorrhage: a prospective, randomized, double-blind phase IIa study. Stroke 38:330–336. 10.1161/01.STR.0000254601.74596.0f 17185636 10.1161/01.STR.0000254601.74596.0f

[7] Carrera E, Schmidt JM, Oddo M, Fernandez L, Claassen J, Seder D, Lee K, Badjatia N, Connolly ES Jr, Mayer SA (2009) Transcranial doppler for predicting delayed cerebral ischemia after subarachnoid hemorrhage. Neurosurgery 65:316–323. 10.1227/01.Neu.0000349209.69973.88 19625911 10.1227/01.NEU.0000349209.69973.88

[8] Chhor V, Le Manach Y, Clarençon F, Nouet A, Daban JL, Abdennour L, Puybasset L, Lescot T (2011) Admission risk factors for cerebral vasospasm in ruptured brain arteriovenous malformations: an observational study. Crit Care 15:R190. 10.1186/cc10345 21831293 10.1186/cc10345 PMC3387632

[9] Dexter F, Hindman BJ (1997) Effect of haemoglobin concentration on brain oxygenation in focal stroke: a mathematical modelling study. Br J Anaesth 79:346–351. 10.1093/bja/79.3.346 9389854 10.1093/bja/79.3.346

[10] Dicpinigaitis AJ, Feldstein E, Shapiro SD, Kamal H, Bauerschmidt A, Rosenberg J, Amuluru K, Pisapia J, Dangayach NS, Liang JW, Bowers CA, Mayer SA, Gandhi CD, Al-Mufti F (2022) Cerebral vasospasm following arteriovenous malformation rupture: a population-based cross-sectional study. Neurosurg Focus 53:E15. 10.3171/2022.4.Focus2277 35901745 10.3171/2022.4.FOCUS2277

[11] Dinc N, Won SY, Eibach M, Quick-Weller J, Konczalla J, Berkefeld J, Seifert V, Marquardt G (2019) Cerebral vasospasm due to arteriovenous malformation-associated hemorrhage: impact of bleeding source and pattern. Cerebrovasc Dis 47:165–170. 10.1159/000500596 31067536 10.1159/000500596

[12] Dorsch NW (1995) Cerebral arterial spasm–a clinical review. Br J Neurosurg 9:403–412. 10.1080/02688699550041403 7546361 10.1080/02688699550041403

[13] Frontera JA, Fernandez A, Schmidt JM, Claassen J, Wartenberg KE, Badjatia N, Connolly ES, Mayer SA (2009) Defining vasospasm after subarachnoid hemorrhage. Stroke 40:1963–1968. 10.1161/STROKEAHA.108.544700 19359629 10.1161/STROKEAHA.108.544700

[14] Gross BA, Du R (2012) Vasospasm after arteriovenous malformation rupture. World Neurosurg 78:300–305. 10.1016/j.wneu.2011.12.090 22381269 10.1016/j.wneu.2011.12.090

[15] Haley EC Jr, Kassell NF, Torner JC, Truskowski LL, Germanson TP (1994) A randomized trial of two doses of nicardipine in aneurysmal subarachnoid hemorrhage. A report of the Cooperative Aneurysm Study. J Neurosurg 80:788–796. 10.3171/jns.1994.80.5.0788 8169616 10.3171/jns.1994.80.5.0788

[16] Inagawa T, Yahara K, Ohbayashi N (2014) Risk factors associated with cerebral vasospasm following aneurysmal subarachnoid hemorrhage. Neurol Med Chir (Tokyo) 54:465–473. 10.2176/nmc.oa.2013-0169 24670311 10.2176/nmc.oa.2013-0169 PMC4533446

[17] Jain A, Huhulea EN, Thorman IB, Beazoglou M, Spirollari E, Sacknovitz A, Soldozy S, Frid I, Thapa S, Schubert MC, Ramakrishnan P, Okafo U, Tyagi R, Gandhi CD, Al-Mufti F (2025) Clinical outcomes of subarachnoid hemorrhage in patients with nicotine dependence: a longitudinal study of 43 315 patients. Neurosurgery. 10.1227/neu.0000000000003695 40891853 10.1227/neu.0000000000003695

[18] Jóhannson H, Siesjö BK (1975) Brain energy metabolism in anesthetized rats in acute anemia. Acta Physiol Scand 93:515–524. 10.1111/j.1748-1716.1975.tb05843.x 1155145 10.1111/j.1748-1716.1975.tb05843.x

[19] Kolias AG, Sen J, Belli A (2009) Pathogenesis of cerebral vasospasm following aneurysmal subarachnoid hemorrhage: putative mechanisms and novel approaches. J Neurosci Res 87:1–11. 10.1002/jnr.21823 18709660 10.1002/jnr.21823

[20] Lasner TM, Weil RJ, Riina HA, King JT Jr., Zager EL, Raps EC, Flamm ES (1997) Cigarette smoking-induced increase in the risk of symptomatic vasospasm after aneurysmal subarachnoid hemorrhage. J Neurosurg 87:381–384. 10.3171/jns.1997.87.3.0381 9285602 10.3171/jns.1997.87.3.0381

[21] Li K, Barras CD, Chandra RV, Kok HK, Maingard JT, Carter NS, Russell JH, Lai L, Brooks M, Asadi H (2019) A review of the management of cerebral vasospasm after aneurysmal subarachnoid hemorrhage. World Neurosurg 126:513–527. 10.1016/j.wneu.2019.03.083 30898740 10.1016/j.wneu.2019.03.083

[22] Liu Z, Khalil RA (2018) Evolving mechanisms of vascular smooth muscle contraction highlight key targets in vascular disease. Biochem Pharmacol 153:91–122. 10.1016/j.bcp.2018.02.012 29452094 10.1016/j.bcp.2018.02.012 PMC5959760

[23] Liu K, Wu L, Yuan S, Wu M, Xu Y, Sun Q, Li S, Zhao S, Hua T, Liu ZJ (2020) Structural basis of CXC chemokine receptor 2 activation and signalling. Nature 585:135–140. 10.1038/s41586-020-2492-5 32610344 10.1038/s41586-020-2492-5

[24] McLaren AT, Marsden PA, Mazer CD, Baker AJ, Stewart DJ, Tsui AK, Li X, Yucel Y, Robb M, Boyd SR, Liu E, Yu J, Hare GM (2007) Increased expression of HIF-1alpha, nNOS, and VEGF in the cerebral cortex of anemic rats. Am J Physiol Regul Integr Comp Physiol 292:R403-414. 10.1152/ajpregu.00403.2006 16973934 10.1152/ajpregu.00403.2006

[25] Ohman J, Servo A, Heiskanen O (1991) Effect of intrathecal fibrinolytic therapy on clot lysis and vasospasm in patients with aneurysmal subarachnoid hemorrhage. J Neurosurg 75:197–201. 10.3171/jns.1991.75.2.0197 1906535 10.3171/jns.1991.75.2.0197

[26] Palchuk MB, London JW, Perez-Rey D, Drebert ZJ, Winer-Jones JP, Thompson CN, Esposito J, Claerhout B (2023) A global federated real-world data and analytics platform for research. JAMIA Open 6:ooad035. 10.1093/jamiaopen/ooad035 37193038 10.1093/jamiaopen/ooad035 PMC10182857

[27] Petridis AK, Fischer I, Cornelius JF, Kamp MA, Ringel F, Tortora A, Steiger HJ (2016) Demographic distribution of hospital admissions for brain arteriovenous malformations in Germany--estimation of the natural course with the big-data approach. Acta Neurochir (Wien) 158:791–796. 10.1007/s00701-016-2727-2 26873715 10.1007/s00701-016-2727-2

[28] Pohjola A, Asikainen A, Kaprio J, Rautalin IM, Niemelä M, Laakso A, Korja M (2025) Sudden prehospital deaths from brain arteriovenous malformations: a population-based study. Neurology 105:e213818. 10.1212/wnl.0000000000213818 40549993 10.1212/WNL.0000000000213818

[29] Rakowski M, Koc NA, Pettersson SD, Zieliński P (2025) Risk factors for cerebral vasospasm following arteriovenous malformation-related hemorrhage: a systematic review and meta-analysis. Neurosurg Rev 48:540. 10.1007/s10143-025-03684-x 40601117 10.1007/s10143-025-03684-x PMC12222334

[30] Rinkel GJ, Feigin VL, Algra A, van Gijn J (2004) Circulatory volume expansion therapy for aneurysmal subarachnoid haemorrhage. Cochrane Database Syst Rev. 10.1002/14651858.CD000483.pub2 15494997 10.1002/14651858.CD000483.pub2 PMC7043358

[31] Rumalla K, Lin M, Ding L, Gaddis M, Giannotta SL, Attenello FJ, Mack WJ (2021) Risk factors for cerebral vasospasm in aneurysmal subarachnoid hemorrhage: a population-based study of 8346 patients. World Neurosurg 145:e233–e241. 10.1016/j.wneu.2020.10.008 33049382 10.1016/j.wneu.2020.10.008

[32] Thomé C, Seiz M, Schubert GA, Barth M, Vajkoczy P, Kasuya H, Schmiedek P (2011) Nicardipine pellets for the prevention of cerebral vasospasm. Acta Neurochir Suppl 110:209–211. 10.1007/978-3-7091-0356-2_38 21125473 10.1007/978-3-7091-0356-2_38

[33] Thorman IB, Schrack JA, Schubert MC (2024) Epidemiology and comorbidities of vestibular disorders: preliminary findings of the AVOCADO study. Otol Neurotol 45:572–579. 10.1097/mao.0000000000004185 38728561 10.1097/MAO.0000000000004185

[34] Thorman IB, Jain A, Mashiach E, Sacknovitz A, Spirollari E, Kaddoura R, Alsaeed R, Schubert MC, Okafo UN, Rosenberg JB, Ramakrishnan P, Mayer SA, Gandhi CD, Al-Mufti F (2025) Impact of acute alcohol intoxication and alcohol dependence on outcomes after subarachnoid hemorrhage. Acta Neurochir 167:231. 10.1007/s00701-025-06639-9 40864201 10.1007/s00701-025-06639-9 PMC12390883

[35] Thorman IB, Sacknovitz A, Wang R, McGoldrick PE, Schubert MC, Mayer SA, Muh CR, Wolf SM (2026) First-line infusion therapies in refractory status epilepticus: A retrospective comparison of outcomes between midazolam and propofol in 7446 patients. Epileptic Disorders. 10.1002/epd2.70174 41525050 10.1002/epd2.70174

[36] Torbey MT, Hauser TK, Bhardwaj A, Williams MA, Ulatowski JA, Mirski MA, Razumovsky AY (2001) Effect of age on cerebral blood flow velocity and incidence of vasospasm after aneurysmal subarachnoid hemorrhage. Stroke 32:2005–2011. 10.1161/hs0901.094622 11546889 10.1161/hs0901.094622

[37] Treggiari MM, Walder B, Suter PM, Romand JA (2003) Systematic review of the prevention of delayed ischemic neurological deficits with hypertension, hypervolemia, and hemodilution therapy following subarachnoid hemorrhage. J Neurosurg 98:978–984. 10.3171/jns.2003.98.5.0978 12744357 10.3171/jns.2003.98.5.0978

[38] Washington CW, Derdeyn CP, Dacey RG, Dhar R, Zipfel GJ (2014) Analysis of subarachnoid hemorrhage using the Nationwide Inpatient Sample: the NIS-SAH severity score and outcome measure: clinical article. Journal of Neurosurgery JNS 121:482–489. 10.3171/2014.4.JNS131100 10.3171/2014.4.JNS131100 24949676

[39] Weir B, Macdonald RL, Stoodley M (1999) Etiology of cerebral vasospasm. Acta Neurochir Suppl 72:27–46. 10.1007/978-3-7091-6377-1_3 10337411 10.1007/978-3-7091-6377-1_3

[40] Xu Y, Ren W, Li Q, Duan C, Lin X, Bi Z, You K, Hu Q, Xie N, Yu Y, Xu X, Hu H, Yao H (2022) LncRNA Uc003xsl.1-mediated activation of the NFκB/IL8 axis promotes progression of triple-negative breast cancer. Cancer Res 82:556–570. 10.1158/0008-5472.Can-21-1446 34965935 10.1158/0008-5472.CAN-21-1446 PMC9359739

[41] Zafar A, Fiani B, Hadi H, Arshad M, Cathel A, Naeem M, Parsons MS, Farooqui M, Bucklin AA, Leone MJ, Baig A, Quadri SA (2020) Cerebral vascular malformations and their imaging modalities. Neurol Sci 41:2407–2421. 10.1007/s10072-020-04415-4 32335778 10.1007/s10072-020-04415-4

